# Distinct survival, optimal combination strategy of immunotherapy, and immunophenotype in uncommon and 20ins EGFR-mut lung adenocarcinoma: a multi-center study

**DOI:** 10.1186/s43556-025-00331-1

**Published:** 2025-10-30

**Authors:** Yiting Sun, Lei Xu, Chaoqiang Deng, Xinyang Du, Yongkui Yu, Yuan Hao, Huijuan Wang, Xin Wang, Yang Zhang, Haiquan Chen

**Affiliations:** 1 Departments of Thoracic Surgery and State Key Laboratory of Genetic Engineering, Fudan University Shanghai Cancer Center, Shanghai, China; 2 Institute of Thoracic Oncology, Fudan University, Shanghai, China; 3 Department of Oncology, Shanghai Medical College, Fudan University, Shanghai, China; 4 Departments of Thoracic Surgery, School of Medicine, Shanghai Chest Hospital, Shanghai Jiao Tong University, Shanghai, China; 5 Department of Thoracic Surgery, National Cancer Center/National Clinical Research Center for Cancer/Cancer Hospital, Chinese Academy of Medical Sciences and Peking Union Medical College, Beijing, China; 6 Department of Medical Oncology, The Affiliated Cancer Hospital of Zhengzhou University & Henan Cancer Hospital, Zhengzhou, Henan Province China; 7 Department of Thoracic Surgery, The Affiliated Cancer Hospital of Zhengzhou University & Henan Cancer Hospital, Zhengzhou, Henan Province China; 8 Department of Clinical Trials Center, Shanxi Province Cancer Hospital/Shanxi Hospital Affiliated to Cancer Hospital, Chinese Academy of Medical Sciences/Cancer Hospital Affiliated to Shanxi Medical University, Taiyuan, Shanxi Province China; 9 Department of Clinical Trials Center, National Cancer Center/National Clinical Research Center for Cancer/Cancer Hospital, Chinese Academy of Medical Sciences and Peking Union Medical College, Beijing, China

**Keywords:** Immunotherapy, EGFR, Uncommon mutations, 20ins mutations, TMB

## Abstract

The efficiency and optimal combination strategies of immune checkpoint inhibitors (ICI) in lung adenocarcinoma (LUAD) patients with epidermal growth factor receptor (EGFR) other mutations apart from 19del/L858R (classical), which account for up to 20% EGFR mutations, remains unclear. In this retrospective multi-center study, a total of 656 EGFR-mut patients receiving ICI alone or with other therapies from several centers were integrated as three independent cohorts and divided into uncommon, classical, and 20ins groups. In all three cohorts, patients in the EGFR uncommon group had longer median progression-free survival (mPFS) than classical or 20ins groups, and longer median overall survival (mOS) than classical group, which were more significant in programmed cell death-ligand 1 (PD-L1) ≥ 1% subgroup. For uncommon group, the mPFS and mOS of ICI alone were similar to ICI plus chemotherapy, but longer than chemotherapy. For 20ins group, the mPFS of ICI plus chemotherapy was longer than ICI alone or chemotherapy. For classical group, ICI plus chemotherapy and anti-angiogenic therapies or plus chemotherapy were found to prolong mPFS than chemotherapy. The tumor mutation burden (TMB) of the uncommon group was relatively higher than classical or 20ins group, with similar PD-L1 expression across them. Uncommon group had more M1 macrophages, less Tregs and M2 macrophages infiltrations than classical or 20ins group. Conclusively, LUAD patients with different EGFR mutations responded to ICI diversely, deserving customed stratification of ICI-based therapies according to specific mutations. EGFR uncommon mutations were linked to relatively higher TMB and heterogeneous immune cell infiltrations.

**Supplementary Information:**

The online version contains supplementary material available at 10.1186/s43556-025-00331-1.

## Introduction

Patients with lung adenocarcinoma (LUAD) harboring EGFR mutations other than exon 19 deletions (19del) and L858R had received insufficient attention, and their treatment options remained limited with suboptimal outcomes. In LUAD patients with uncommon EGFR mutations (e.g., S768I, G719X, L861Q), although afatinib [1] and osimertinib [2, 3] demonstrated efficacy, acquired resistance inevitably developed, leaving limited subsequent therapeutic options. For LUAD patients with EGFR 20 exons insertions (20ins) mutations, the efficiency of traditional chemotherapies and tyrosine kinase inhibitors (TKI) was unsatisfactory. Previous studied reported that mPFS of first line chemotherapy, TKI, or ICI monotherapy in EGFR 20ins patients ranged 4.5–6.4, 0.7–3.7, or 3.1–4.3 months, respectively [4–8]. Whether immune checkpoint inhibitors (ICI)—a promising therapeutic approach in lung cancer [9–12]—confer survival benefits in patients with uncommon EGFR mutations or 20ins remained unclear and warranted further investigation. Tumor mutation burden (TMB), programmed cell death ligand 1(PD-L1) expression, and the infiltrations of some vital cell types in tumor immune microenvironments were considered close to response to ICI [13–17], while how they regulated patients with EGFR 20ins mutations responding to ICI were still unclear.

Moreover, previous studies have indicated strong heterogeneity across EGFR mutation subtypes: compared with EGFR 19del, patients with EGFR L858R mutations responded to ICI better and similarly to wild-type patients [18]. Lung cancer patients with T790M-negative EGFR mutations were more likely to benefit from nivolumab treatment [19]. Hence, we conjectured that other EGFR mutations, including S768I, G719X, L861Q, and 20ins, might also respond to ICI diversely, which has not been reported yet.

Besides, previous IMpower150 [20], ORIENT-31 [21], and ATTLAS [22] trials all confirmed ICI plus chemotherapy plus anti-angiogenic therapies prolonged survival of EGFR-mut patients. However, 19del/L859R mutations accounted for 73% [20], 94.2% [21], and 96.6% [22] in these trials. Several small-sample studies also analyzed the efficacy of immunotherapy in EGFR-mut patients, with 19del/L858R mutations accounting for 100% [23], 97% [24], 93% [25], 92% [26], and 79% [27], respectively. In all these studies, specific analysis regarding EGFR other mutations was absent. Hence, whether the findings of these studies are applicable to patients with other EGFR mutations is unclear. Considering other EGFR mutation subtypes accounting for over 20% of all patients [1, 28, 29], it is necessary to identify an optimal combination strategy specific to these patients.

Taken together, whether response to ICIs among patients with different EGFR mutations was different was still unclear. The optimal combination strategy for each mutation subtypes was uncertain. Here, based on three cohorts comprising 656 EGFR-mut patients receiving ICI from Fudan University Shanghai Cancer Center and other centers, we aimed to compare the response in patients with EGFR uncommon and 20ins mutations and identified an optimal combination strategy specific to each mutation subtype. The tumor mutation burden (TMB), PD-L1, and tumor immune cell infiltrations were also analyzed across these EGFR mutation subtypes, to explore the potential underlying mechanisms.

## Results

### Patients with EGFR uncommon mutation benefit more from immune checkpoint inhibitors than those with classical or 20ins mutations

**Fig. 1:**
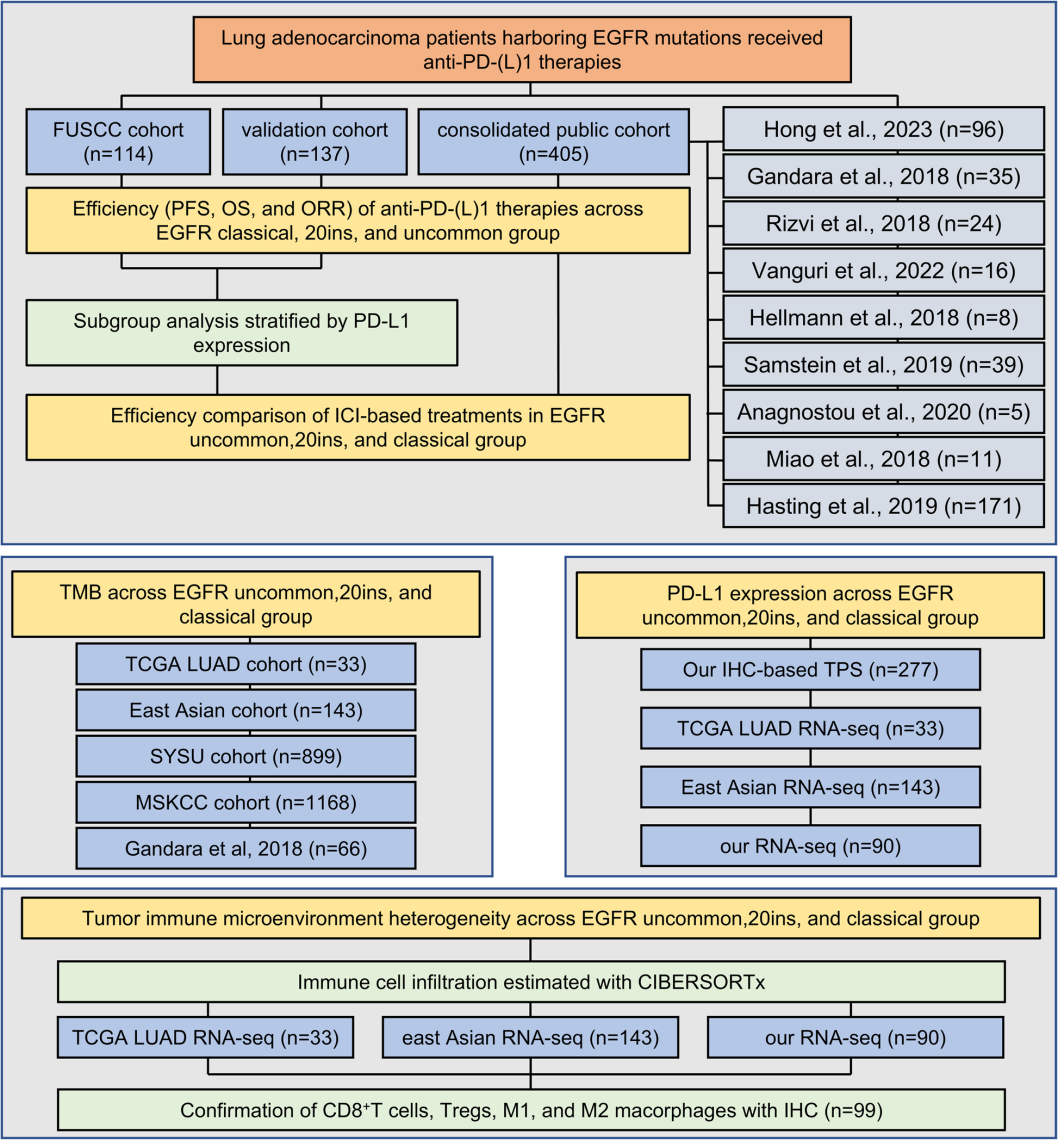
The analysis schema of this study

To compare the responses of patients with different EGFR mutations to ICI, 114 EGFR-mut LUAD patients receiving ICI at Fudan University Shanghai Cancer Center (FUSCC) were retrospectively collected as the discovery cohort (Table S1). The patients were stratified into three groups according to their EGFR mutations, namely classical group, exon 19 deletions (19del) or exon 21 L858R mutations; 20ins group, exon 20 insertions; uncommon group, EGFR other mutations except for 19del, L858R, and 20ins. A total of 137 patients with advanced EGFR-mut LUAD and receiving ICI at Henan Cancer Hospital and Shanxi Province Cancer Hospital were retrospectively analyzed as the validation cohort (Table S2). The public cohort comprised nine independent open-access cohorts containing 405 LUAD patients with EGFR mutations who received ICI (Table S3). The analysis schema was shown in Fig. 1.

**Fig. 2:**
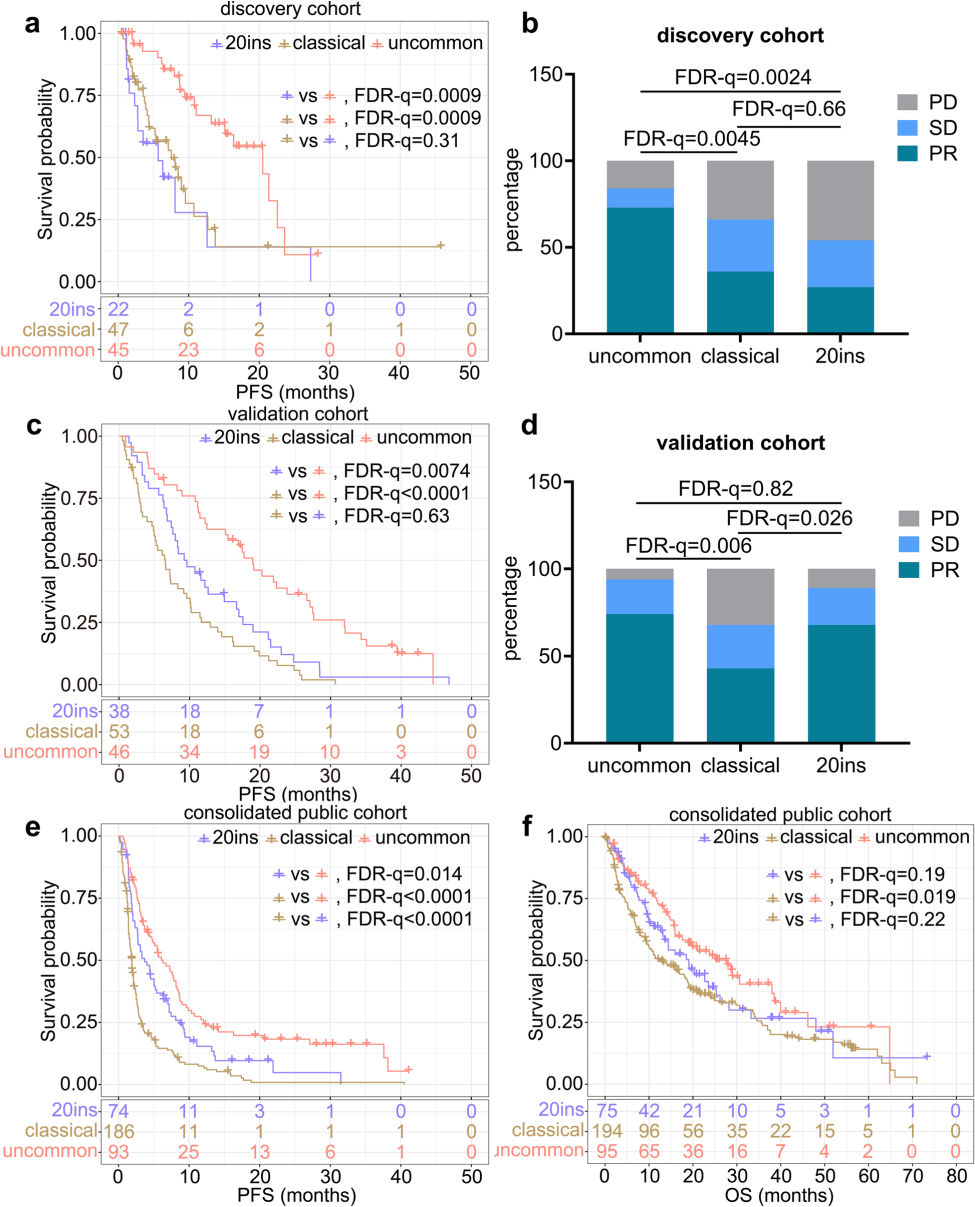
Heterogenous responses of EGFR mutations subtypes to ICI. **a**-**b** PFS (**a**) and PR rate (**b**) across EGFR mutation subtypes in the discovery cohort. **c**-**d** PFS (**c**) and PR rate (**d**) across EGFR mutation subtypes in the validation cohort. **e**-**f** PFS (**e**) and OS (**f**) across EGFR mutation subtypes in the consolidated public cohort

First, in the discovery cohort, median progression-free survival (mPFS, calculated in months) of all three groups were compared. The mPFS of the uncommon group was longer than that of the 20ins (20.50 vs. 5.67, hazard ration (HR) = 0.38, False Discovery Rate q value (FDR-q) = 0.0009; Fig. 2a) and classical groups (20.50 vs. 7.53, HR = 0.37, FDR-q = 0.0009; Fig. 2a). No significant difference was observed between the 20ins and classical groups (5.67 vs. 7.53, HR = 0.82, FDR-q = 0.31; Fig. 2a). The rate of partial response (PR) in the uncommon group was higher than that in the classical (73% vs. 36%, FDR-q = 0.0045; Fig. 2b) and 20ins (73% vs. 27%, FDR-q = 0.0024; Fig. 2b) groups. The PR rate was similar between the classical and 20ins groups (36% vs. 27%, FDR-q = 0.66; Fig. 2b). In the validation cohort, the mPFS of the uncommon group was longer than that of the 20ins (18.97 vs. 9.43, HR = 0.50, FDR-q = 0.0074; Fig. 2c) or classical group (18.97 vs. 6.57, HR = 0.37, FDR-q < 0.0001; Fig. 2c). No significant difference was observed between the 20ins and classical groups (9.43 vs. 6.57, HR = 0.70, FDR-q = 0.063; Fig. 2c). In addition, the PR rate of the uncommon group was higher than that of the classical group (74% vs. 43%, FDR-q = 0.006; Fig. 2d), whereas the PR rate of the 20ins and uncommon (68% vs. 74%, FDR-q = 0.82; Fig. 2d) was similar. The PR rate in the 20ins group was higher than that in the classical group (68% vs. 43%, FDR-q = 0.026; Fig. 2d).

The consolidated public cohort aggregated data from nine independent open-access studies, encompassing 405 EGFR-mutant LUAD patients treated with ICI. In this cohort, the mPFS (6.30 vs. 3.71, HR = 0.66, FDR-q = 0.014; Fig. 2e) of the uncommon was longer than 20ins group, with a tendency of prolonged median overall survival (mOS, calculated in months) (27.73 vs. 19.07, HR = 0.76, FDR-q = 0.19; Fig. 2f). The mPFS (6.30 vs. 1.87, HR = 0.41, FDR-q < 0.0001; Fig. 2e) and mOS (27.73 vs. 12.42, HR = 0.64, FDR-q = 0.019; Fig. 2f) of the uncommon group were longer than those of the classical group. In addition, the mPFS (3.71 vs. 1.87, HR = 0.57, FDR-q < 0.0001; Fig. 2e) of the 20ins group was longer than those of the classical group, with a tendency of prolonged mOS (19.07 vs. 12.42, HR = 0.77, FDR-q = 0.22; Fig. 2f).

To further confirm whether the survival differences were ICI specific, the recurrence-free survival (RFS) and OS of 3436 patients from FUSCC, harboring EGFR mutations who underwent radical resection were analyzed. RFS (*p* = 0.25; Fig. S1a) and OS (*p* = 0.44; Fig. S1b) across all three subtypes were similar.

Collectively, across all three cohorts, patients with uncommon EGFR mutations treated with ICIs demonstrated superior objective response rate (ORR), prolonged PFS, and extended OS relative to those with classical mutations, and higher PR rate in the discovery cohort and longer PFS than 20ins patients in all three cohorts.

### Response heterogeneity was more significant in PD-L1 ≥ 1% or not accessible subgroups

**Fig. 3:**
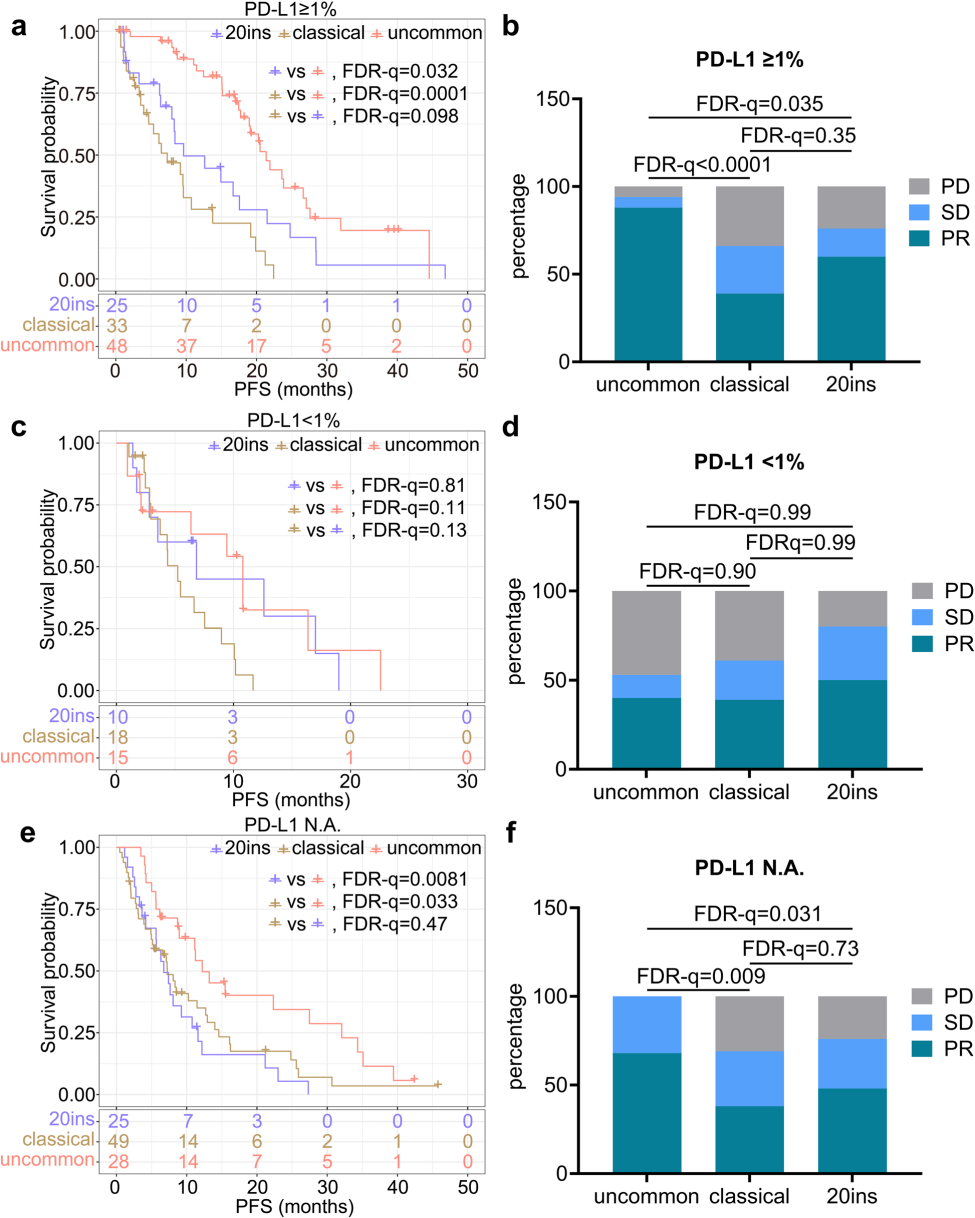
Response heterogeneity was more significant in PD-L1 ≥ 1% and N.A. subgroups. **a**-**b** PFS (**a**) and PR rate (**b**) of PD-L1 ≥ 1% subgroup across EGFR mutation subtypes in our cohort. **c**-**d** PFS (**c**) and PR rate (**d**) of PD-L1 < 1% subgroup across EGFR mutation subtypes in our cohort. **e**-**f** PFS (**e**) and PR rate (**f**) of PD-L1 N.A. subgroup across EGFR mutation subtypes in our cohort

Next, the survival diversity of the EGFR mutation subtypes within different PD-L1 expression levels was explored. In this section, patients from the discovery cohort and the validation cohort were pooled due to the limited number of patients in each PD-L1 expression subgroup. In the PD-L1 ≥ 1% subgroup, mPFS of the uncommon group was longer than that of the classical (21.37 vs. 7.30, HR = 0.25, FDR-q = 0.0001; Fig. 3a) or 20ins group (21.37 vs. 9.63, HR = 0.45, FDR-q = 0.032; Fig. 3a). The mPFS of the classical and 20ins groups were similar (7.30 vs. 9.63, HR = 1.32, FDR-q = 0.098; Fig. 3a). In addition, the PR rate in the uncommon group was higher than that in the classical (88% vs. 39%, FDR-q < 0.0001; Fig. 3b) or 20ins (88% vs. 60%, FDR-q = 0.035; Fig. 3b) group. No significant difference was observed between the classical and 20ins groups (39% vs. 60%, FDR-q = 0.35; Fig. 3b).

In the PD-L1 < 1% subgroup, the mPFS of uncommon, classical, and 20ins groups were similar (all FDR-q ≥ 0.05; Fig. 3c). The PR rates across the three subgroups were also similar (all FDR-q ≥ 0.05; Fig. 3d).

In the PD-L1 expression not accessible (N.A.) subgroup, the mPFS of the uncommon group was longer than that of the classical (12.20 vs. 7.23, HR = 0.55, FDR-q = 0.033; Fig. 3e) or 20ins group (12.20 vs. 6.73, HR = 0.55, FDR-q = 0.0081; Fig. 3e), while the mPFS of the classical and 20ins groups were similar (7.23 vs. 6.73, HR = 0.93, FDR-q = 0.47; Fig. 3e). In addition, the PR rate of uncommon group was higher than that in the classical (68% vs. 38%, FDR-q = 0.009; Fig. 3f) or 20ins group (68% vs. 48%, FDR-q = 0.031; Fig. 3f). There was no significant difference between the 20ins and classical (48% vs. 38%, FDR-q = 0.73; Fig. 3f).

Taken together, patients with EGFR uncommon mutations had better response to ICI than those with classical or 20ins mutations in PD-L1 ≥ 1%, or N.A. but not the PD-L1 < 1% subgroup. Patients with EGFR 20ins mutations responded to ICI similarly to those with classical mutations in all PD-L1 expression subgroups.

### ICI alone or plus chemotherapy was effective in patients with EGFR uncommon mutations

**Fig. 4:**
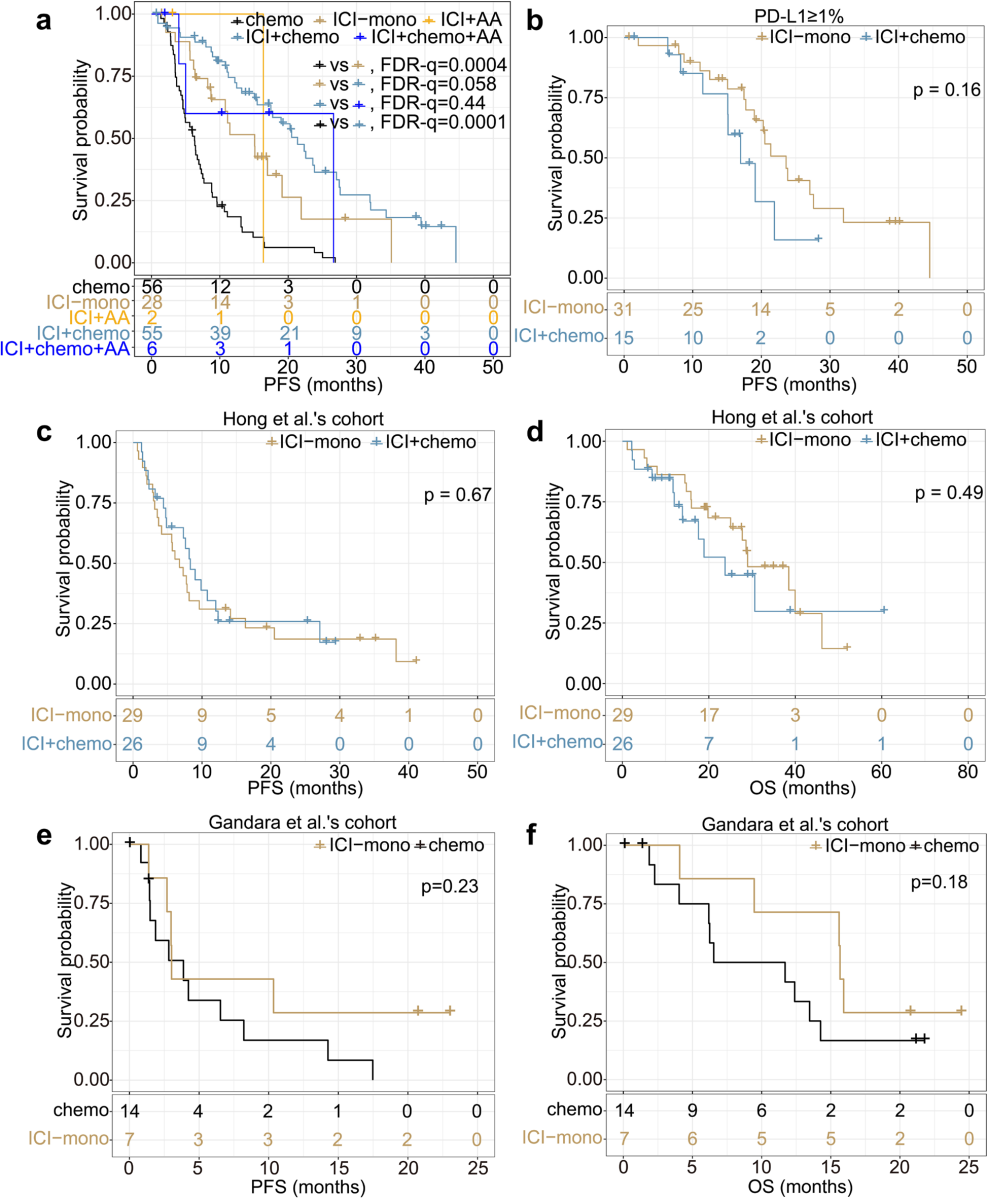
Efficiency comparison of different treatments in uncommon group. **a** PFS of chemotherapy and ICI-based therapies in our cohort. **b** PFS of ICI + chemo vs ICI-mono in PD-L1 ≥ 1% subgroup in our cohort. **c**-**d** PFS (**c**) and OS (**d**) of ICI + chemo vs ICI-mono in Hong et al.’s cohort. **e**-**f** PFS (**e**) and OS (**f**) of chemo vs ICI-mono in Gandara et al.’s cohort

The optimal combination strategy of ICI has not been clarified for patients with EGFR uncommon mutations. All 91 patients from the discovery and the validation cohorts were categorized into four groups, namely ICI monotherapy (ICI-mono), ICI plus chemotherapies (ICI + chemo), ICI plus anti-angiogenic therapies (ICI + AA), ICI plus chemotherapies and anti-angiogenic therapies (ICI + chemo + AA). Fifty-six of 91 patients received chemotherapies before ICI, were taken as self-control. The mPFS of the ICI + chemo (21.37 vs. 6.30, HR = 0.27, FDR-q = 0.0001; Fig. 4a) or ICI-mono (15.10 vs. 6.30, HR = 0.39, FDR-q = 0.0004; Fig. 4a) group was longer than that of the chemo group. The mPFS of ICI + chemo group was tending to be higher than the ICI-mono group (21.37 vs. 15.10, HR = 0.57, FDR-q = 0.058; Fig. 4a). The mPFS in the ICI + chemo + AA group was similar to that of the ICI + chemo group (21.37 vs. 26.63, HR = 1.57, FDR-q = 0.44; Fig. 4a). Patients in the ICI + AA group were not analyzed because of the limited number of patients included (*n* = 3). Moreover, in the PD-L1 ≥ 1% subgroup, mPFS of ICI + chemo and ICI-mono groups were not different (23.60 vs. 16.97, HR = 0.56, *p* = 0.16; Fig. 4b).

Furthermore, in the Hong et al.’s cohort, there was no significant difference in mPFS (8.30 vs. 6.73, HR = 0.88, *p* = 0.67; Fig. 4c) or mOS (23.75 vs. 28.97, HR = 1.30, *p* = 0.49; Fig. 4d) between ICI + chemo and ICI-mono groups. In the Gandara et al.’s cohort, there was no significant difference in the mPFS between the atezolizumab and docetaxol groups (3.02 vs. 3.88, HR = 1.82, *p* = 0.23; Fig. 4e); however, there was a tendency for the atezolizumab group having longer mOS than the docetaxol group (15.67 vs. 9.12, HR = 0.50, *p* = 0.18; Fig. 4f).

In conclusion, the effectiveness of the ICI + chemo and ICI-mono groups was similar, and both of their mPFS were longer than that of the chemotherapy group. No additional benefits of anti-angiogenic therapies based on ICI plus chemotherapy were observed.

### ICI plus chemotherapy, but not alone, was effective in patients with EGFR 20ins mutations

**Fig. 5:**
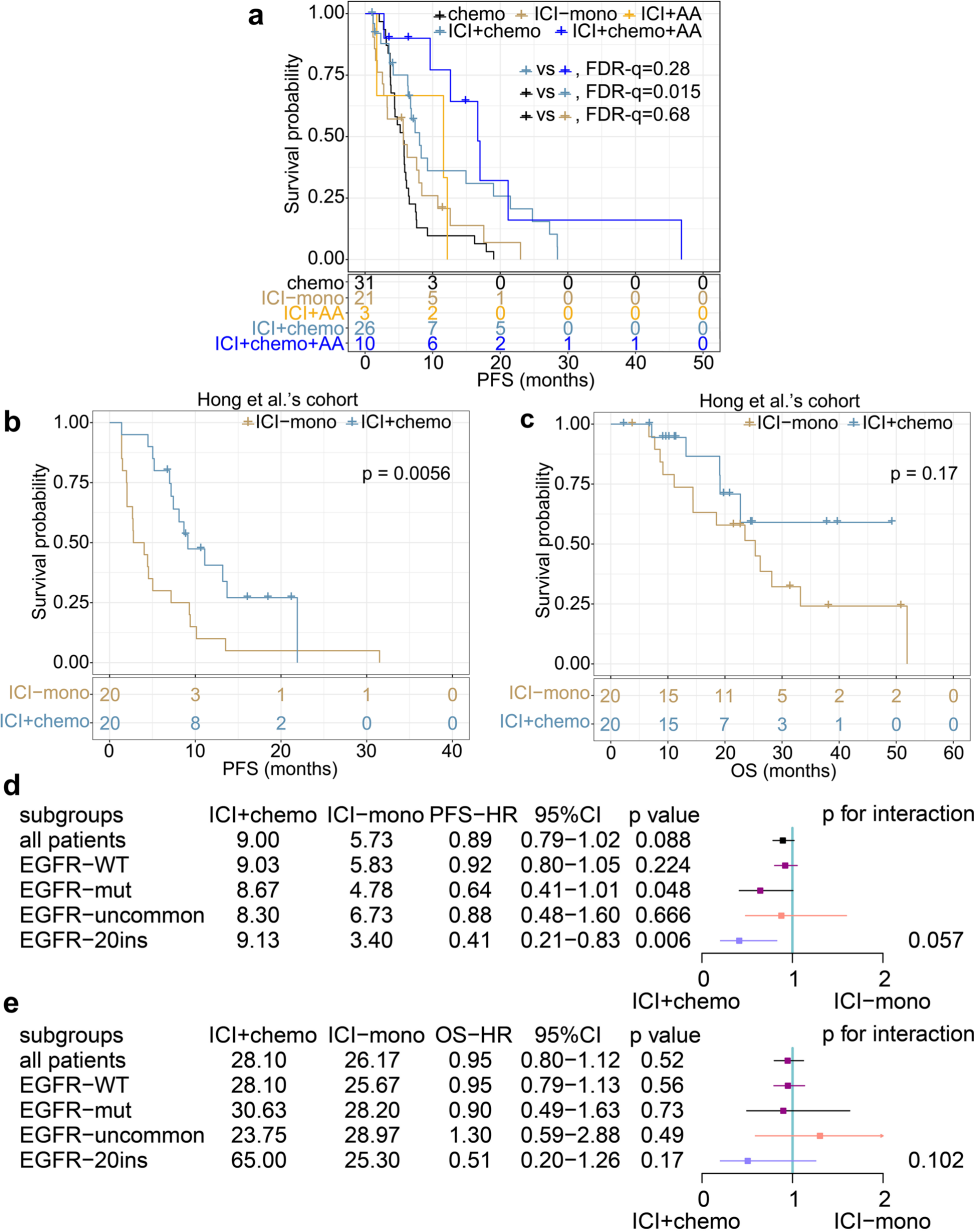
Efficiency comparison of different treatments in 20ins group. **a** PFS of chemotherapy and ICI-based therapies in our cohort. **b**-**c** PFS (**b**) and OS (**c**) of ICI + chemo vs ICI-mono in Hong et al.’s cohort. **d**-**e** Forest plot of ICI + chemo vs ICI-mono in term of PFS (**d**) and OS (**e**) in Hong et al.’s cohort

The optimal combination strategy of ICI in the 20ins subgroup remains to be clarified. All 60 patients from the discovery and the validation cohorts were categorized into four groups as mentioned above, with chemo-PFS of 31 patients as self-control. Compared with the chemo group, the mPFS of the ICI-mono group was similar (5.67 vs. 5.67, HR = 0.99, FDR-q = 0.68; Fig. 5a), while the mPFS of the ICI + chemo group was longer (8.06 vs. 5.67, HR = 0.48, FDR-q = 0.015; Fig. 5a). The mPFS in the ICI + chemo + AA group was similar to that of the ICI + chemo group (16.67 vs. 8.06, HR = 0.58, FDR-q = 0.28; Fig. 5a). Patients in the ICI + AA group were not analyzed because of the limited number of patients included (*n* = 2).

In the Hong et al.’s cohort, the mPFS of ICI + chemo group was longer than that of the ICI-mono group (9.13 vs. 3.40, HR = 0.41, *p* = 0.0056; Fig. 5b), with a tendency of prolonged mOS (over 49.23 vs. 25.30, HR = 0.50, *p* = 0.17; Fig. 5c). More importantly, the *p*-values for interaction for analyzing PFS and OS were 0.057 (Fig. 5d) and 0.102 (Fig. 5e), respectively, indicating that ICI + chemo specifically prolonged PFS and OS in the 20ins, but not uncommon group.

Taken together, for patients with EGFR 20ins mutations, ICI + chemotherapy was better than chemotherapy, with ICI-mono similar to chemotherapy. ICI + chemotherapy + AA might not bring more survival benefits and needed more validation and caution owing to the adverse effects of combinations.

### ICI plus chemotherapy, but not alone or with anti-angiogenic therapies was effective in patients with EGFR classical mutations

**Fig. 6:**
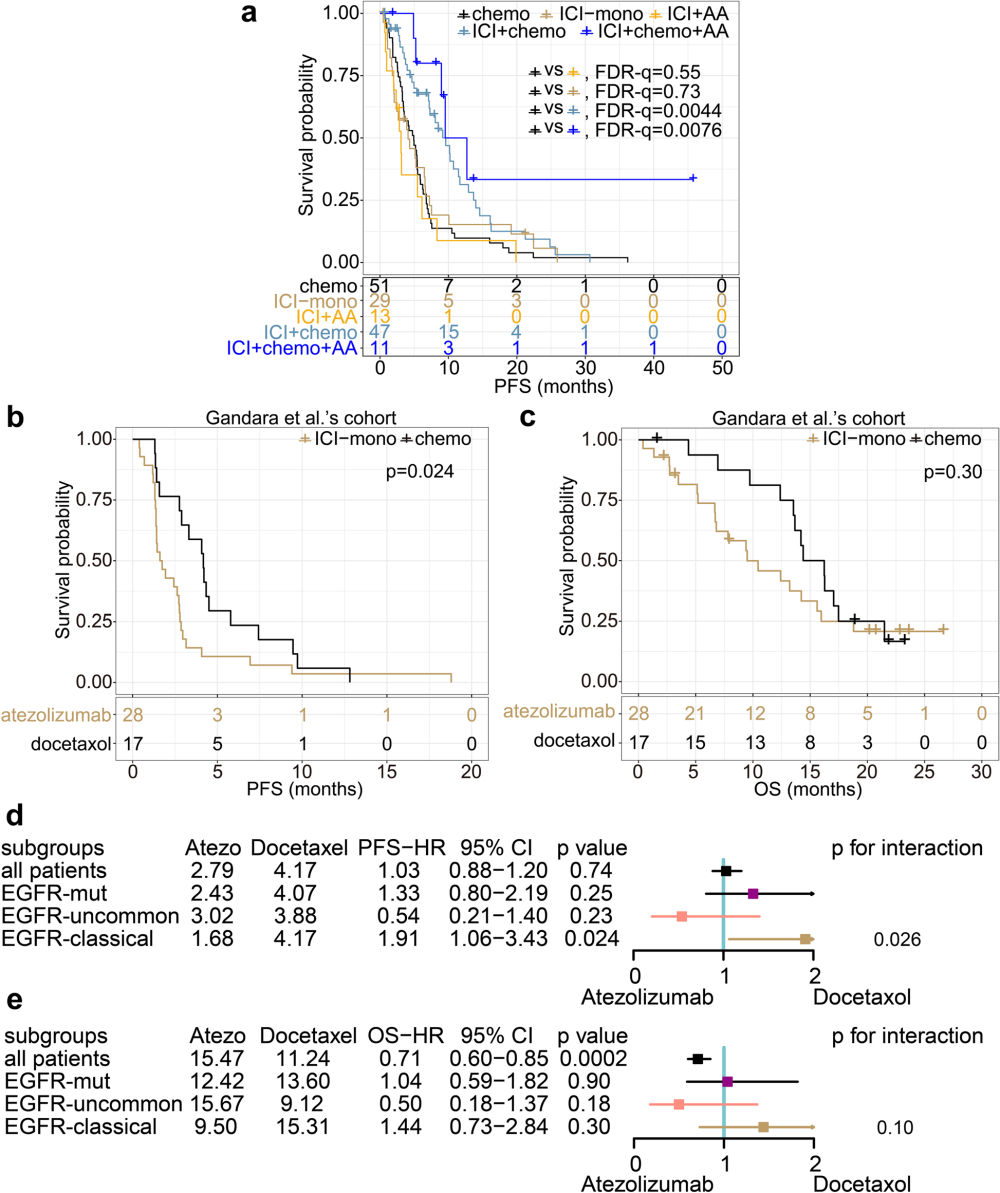
Efficiency comparison of different treatments in classical group. **a** PFS of chemotherapy and ICI-based therapies in our cohort. **b**-**c** PFS (**b**) and OS (**c**) of chemo vs ICI-mono in Gandara et al.’s cohort. **d**-**e** Forest plot of chemo vs ICI-mono in term of PFS (**d**) and OS (**e**) in Hong et al.’s cohort

Previous studies have confirmed that ICI + chemo + AA is superior to other ICI-based therapies [20, 21]. However, considering cost and accessibility, other options are expected. From the discovery and the validation cohort, 100 patients were categorized into four groups as mentioned above, with chemo-PFS of 51 patients as self-control. The ICI + chemo or ICI + chemo + AA group had longer mPFS than chemo (11.12 vs. 4.93, HR = 0.26, FDR-q = 0.0076; 9.60 vs. 4.93, HR = 0.53, FDR-q = 0.0044; Fig. 6a). While mPFS of ICI-mono or ICI + AA were similar to chemo (4.10 vs. 4.93, HR = 1.08, FDR-q = 0.73; 3.07 vs. 4.93, HR = 1.30, FDR-q = 0.55; Fig. 6a).

In the Gandara et al.’s cohort, the mPFS of the atezolizumab group was shorter than that of the docetaxol group (1.68 vs. 4.17, HR = 1.91, *p* = 0.024; Fig. 6b). The median OS was similar between the two groups (9.50 vs. 15.31, HR = 1.44, *p* = 0.30; Fig. 6c); the *p*-values for interaction for analyzing PFS and OS were 0.026 and 0.10 (Fig. 6d-e), indicating that atezolizumab specifically shortened PFS in classical, but not uncommon patients.

Taken together, except for ICI + chemo + AA, ICI + chemo was also an effective alternative to EGFR classical mutations patients, while ICI + AA or ICI monotherapy was not.

In addition, overview of survival across the three mutation groups for each treatment strategy were summarized in Table S4.

### Associations of EGFR mutation subtypes and TMB or PD-L1 expression

**Fig. 7:**
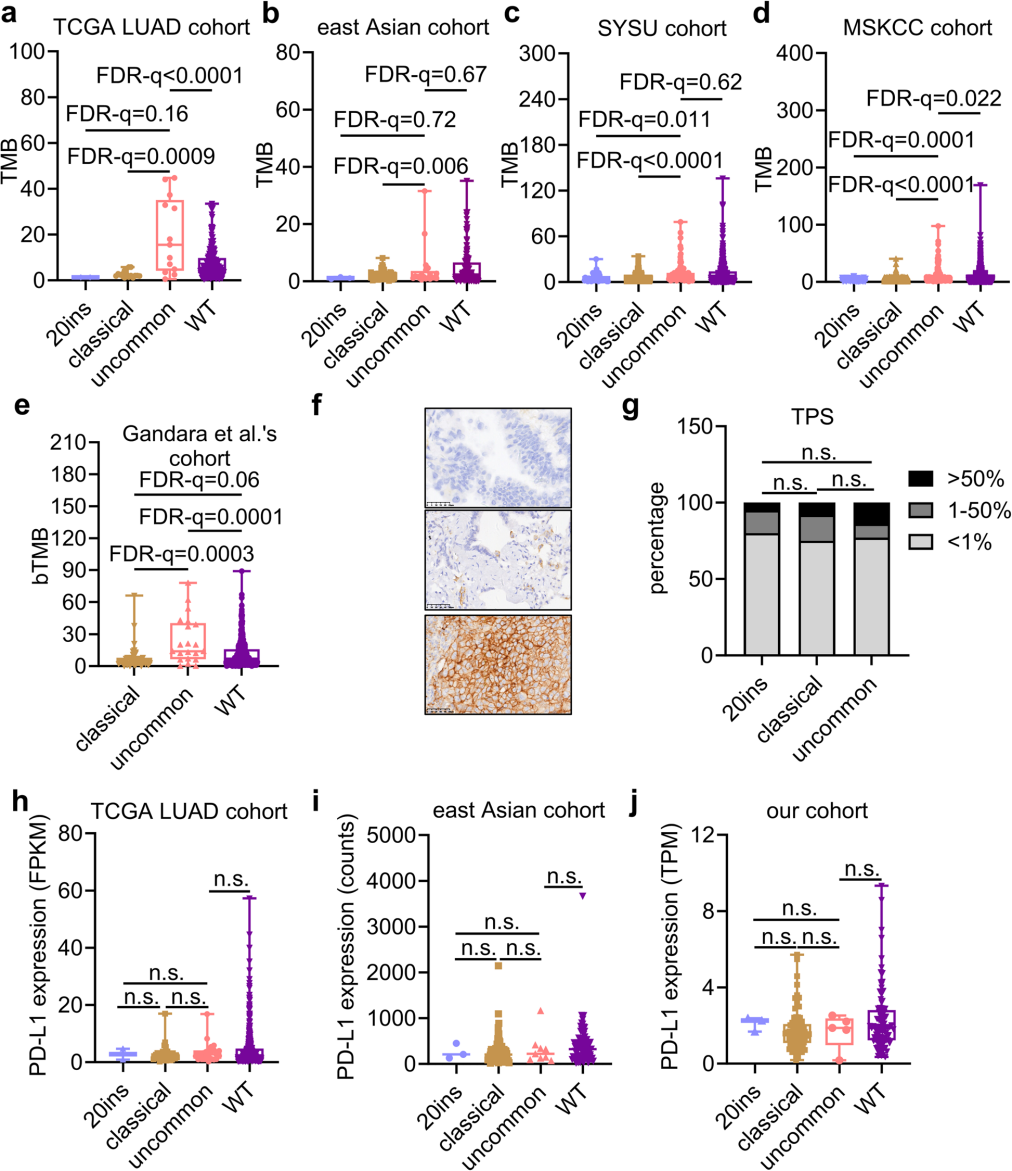
TMB or PD-L1 expression across EGFR mutation subtypes. **a**-**e** TMB or bTMB across EGFR mutation subtypes in TCGA LUAD (**a**), east Asian (**b**), SYSU (**c**), MSKCC (**d**) and Gandara et al.’ cohort (**e**). **f** Representative IHC figures of PD-LD TPS < 1% (upper), 1–50% (middle), and ≥ 50% (bottom). **g** PD-L1 TPS in 277 samples with EGFR mutation subtypes. **h**-**j** PD-L1 RNA expression across EGFR mutation subtypes in TCGA LUAD (**h**), est Asian (**i**), and our cohort (**j**). n.s., not significant

TMB and PD-L1 expression were linked to the response to ICI. Hence, differences in TMB and PD-L1 expression across EGFR mutation subtypes were investigated. In the TCGA LUAD cohort, the TMB of the uncommon group was relatively higher than that of the classical (19.42 vs. 2.69, FDR-q = 0.0009; Fig. 7a) and wild-type (WT) groups (19.42 vs. 7.69, FDR-q < 0.0001; Fig. 7a), with a tendency of being relatively higher than the 20ins group (19.42 vs. 1.33, *p* = 0.16; Fig. 7a). In the east Asian cohort, the TMB of the uncommon group was similar to that of the 20ins (4.24 vs. 1.14, FDR-q = 0.72; Fig. 7b) and WT (4.24 vs. 4.92, *p* = 0.67; Fig. 7b) groups, but relatively higher than the classical group (4.24 vs. 2.05, FDR-q = 0.006; Fig. 7b). In the SYSU cohort, the TMB of the uncommon group was relatively higher than that of the 20ins (11.17 vs. 5.60, FDR-q = 0.011; Fig. 7c) or classical (11.17 vs. 5.27, FDR-q < 0.0001; Fig. 7c) group, but similar to WT (11.17 vs. 10.49, FDR-q = 0.62; Fig. 7c). In the MSKCC cohort, the TMB of the uncommon group was relatively higher than that of the 20ins (9.85 vs. 3.60, FDR-q = 0.0001; Fig. 7d), classical (9.85 vs. 4.39, FDR-q < 0.0001, Fig. 7d), and even WT (9.85 vs. 8.32, FDR-q = 0.022; Fig. 7d) groups. In Gandara et al.’s cohort, the blood TMB (bTMB) of the uncommon group was relatively higher than that of the classical (23.90 vs. 7.71, FDR-q = 0.0003; Fig. 7e) and WT groups (23.90 vs. 11.01, FDR-q = 0.0001; Fig. 7e). While the bTMB of classical group was tending to be lower than WT group (7.71 vs 11.01, FDR-q = 0.06; Fig. 7e). Taken together, the TMB or bTMB of the uncommon group was relatively higher than that of the 20ins group (except for the TGGA LUAD and east Asian cohorts, possibly owing to limited samples), and the classical group, similar to or even higher than WT.

PD-L1 expression was compared with the tumor proportion score (TPS) based on the IHC analysis of 277 samples, consisting of 102 samples with 20ins, 97 with classical mutations, and 78 with uncommon mutations. Representative figures of TPS < 1% (Fig. 7f, upper), 1–50% (Fig. 7f, middle), and ≥ 50% (Fig. 7f, bottom) were displayed. The TPS across the EGFR mutation subtypes was similar (all FDR-q ≥ 0.05; Fig. 7g). In the TCGA LUAD cohort (Fig. 7h), east Asian cohort (Fig. 7i), and our own cohort (Fig. 7j), PD-L1 RNA expression in the uncommon group was similar to that in the 20ins, classical, and WT groups (all FDR-q ≥ 0.05; Fig. 7h-j). In conclusion, PD-1 expression was similar across EGFR mutation subtypes.

### Tumor immune microenvironment diversity of EGFR mutation subtypes

**Fig. 8:**
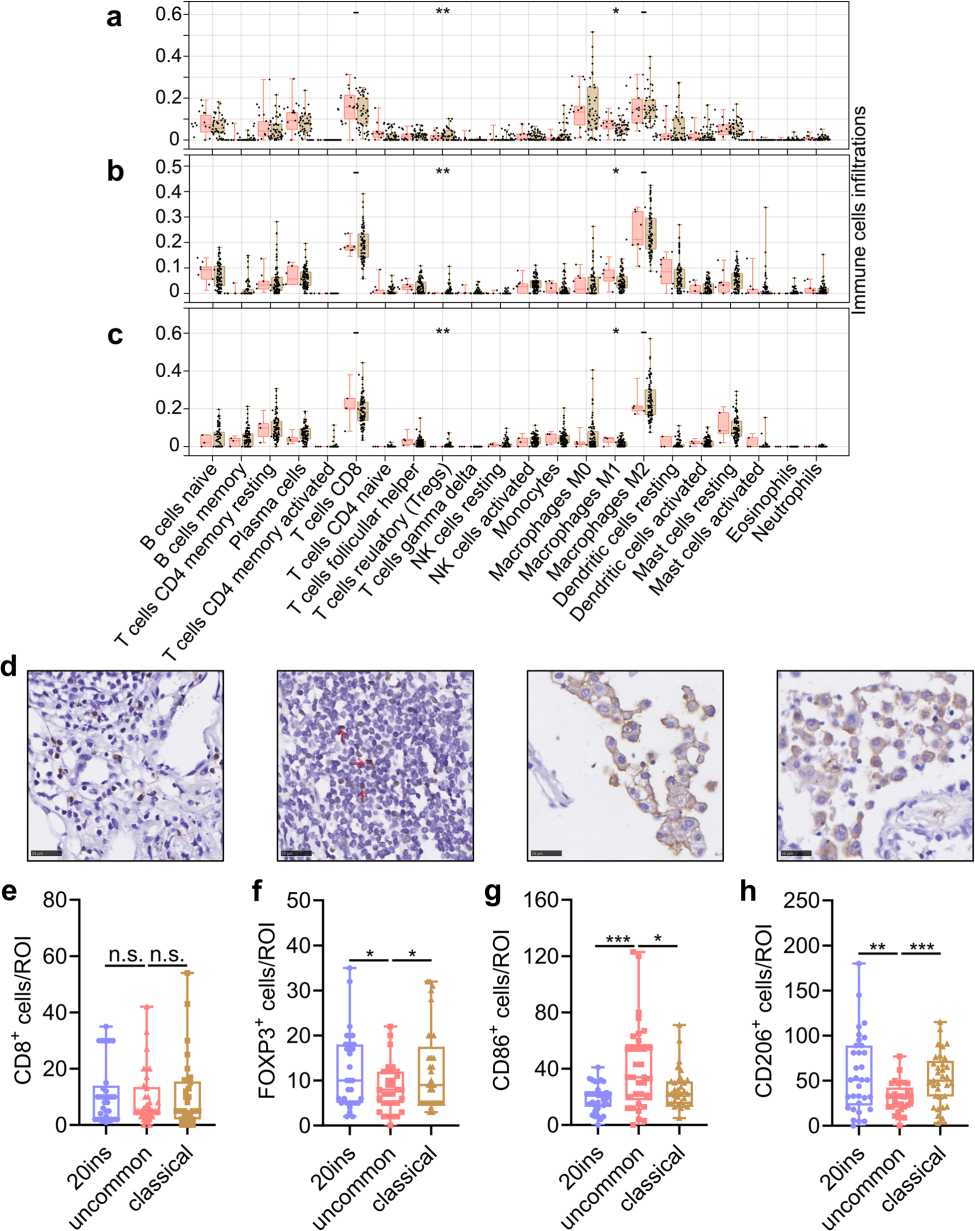
Tumor immune microenvironment heterogeneity across EGFR mutation subtypes. **a**-**c** Estimation of immune cell infiltration across EGFR mutation subtypes based on CIBERSORTx with RNA sequencing data from TCGA LUAD (**a**), east Asian (**b**), and our cohort (**c**). **d** Representative IHC figures of CD8^+^, FOPX3^+^, CD86^+^, and CD206^+^ cells (from left to right). **e**-**h** Comparison of CD8^+^ (**e**), FOPX3^+^ (**f**), CD86^+^ (**g**), and CD206^+^ (**h**) cells infiltration based on IHC. n.s., not significant; ***, FDR-q < 0.001; **, FDR-q < 0.01; *, FDR-q < 0.05

Tumor-infiltrating immune cells were involved in the response to ICI. Here, the infiltrations of 22 types of immune cells, including CD8^+^ T cells, regulatory T cells (Tregs), and type 1/2 macrophages (M1/M2), were estimated using CIBERSORTx, based on RNA-seq data from the TCGA LUAD (Fig. 8a), east Asia (Fig. 8b), and our own cohort (Fig. 8c). In all the three cohorts, compared with classical group, samples with EGFR uncommon mutations had less Tregs (all FDR-q < 0.01; Fig; 8a-c) and more M1 macrophages (all FDR-q < 0.05; Fig. 8a-c), while CD8^+^ T and M2 macrophages (all FDR-q ≥ 0.05; Fig. 8a-c) were similar between the two groups. Immune cell infiltrations in 20ins group were not analyzed due to the limited samples (*n* = 2 or 3).

To confirm the above findings, Tregs, CD8^+^ T, M1, and M2 macrophage infiltration in 99 samples (33 in each group) harboring EGFR mutations were assessed using immunohistochemical staining, with FOXP3, CD8A, CD86, and CD206 as the respective markers (Fig. 8d). No significant difference was observed when comparing CD8^+^ T cells across these subtypes (both FDR-q ≥ 0.05; Fig. 8e). FOXP3^+^ Treg infiltration in the uncommon group was less than that in the 20ins (FDR-q = 0.046; Fig. 8f) or classical (FDR-q = 0.031; Fig. 8f) group. Uncommon group had more CD86^+^ M1 macrophage infiltration than those in 20ins (FDR-q = 0.0004; Fig. 8g) or classical (FDR-q = 0.0085, Fig. 8g) group. CD206^+^ M2 infiltration in the uncommon group was less than that in the 20ins (FDR-q = 0.0012; Fig. 8h) or classical (FDR-q = 0.0006; Fig. 8h) group.

Taken together, there was greater infiltration of M1 macrophages and fewer Tregs and M2 macrophages in the uncommon group than in the 20ins or classical group, while CD8^+^ T cell infiltration was similar.

## Discussion

While ICI have revolutionized the treatment of non-small cell lung cancer (NSCLC), prior studies indicate limited benefit in patients with EGFR-mutant lung adenocarcinoma [9–12]. However, patients harboring different mutation subtypes were reported to responded to ICI differently [18, 19], indicating huge response heterogeneity across EGFR mutation subtypes. To our knowledge, this is the first study demonstrating that patients harboring uncommon EGFR mutations exhibit superior responses to ICI compared to those with 20ins or 19del/L858R alterations.

The differences were more pronounced in PD-L1 ≥ 1% subgroup. This observation may stem from 19del/L858R alterations’ poor response to all PD-L1 expression, but uncommon mutations’ response to higher PD-L1 expression. The heterogeneous responses to ICIs across EGFR mutation subtypes underscore the necessity of stratifying treatment strategies in clinical practice and trial design for EGFR-mutant patients.

To further prove the necessity of stratification, we also determined the optimal ICI strategy for each group. For patients with uncommon EGFR mutations, additional chemotherapy did not add survival benefits to ICI in either our or public cohort, suggesting additional chemotherapy may be unnecessary and may bring extra adverse events. In a previous study, mPFS of afatinib was 10.7 months and mOS was 19.4 months [1]. In the UNICORN study, the mPFS of first-line osimertinib in patients with uncommon EGFR mutations was 9.5 months, while mOS was 24.5 months [2]. The mPFS of first-line osimertinib was 8.2 months in a phase II study [3]. In our present study, mPFS of ICI plus chemotherapy or ICI monotherapy was 21.37 or 15.10 months, respectively. In Hong et al.’s cohort, the mOS of patients who received ICI plus chemotherapy or ICI mono was 23.75 or 28.97 months, respectively. These data suggest that ICIs may represent a quite effective alternative. Therefore, ICI plus chemo or alone, was promising treatments among those with EGFR uncommon mutations, especially after acquired resistance to TKI, but needing prospective and head-to-head comparisons to validate. However, considering the association of PD-L1 expression and response to ICI, testing of PD-L1 expression and/or TMB is still necessary for stratification. Unfortunately, due the limited sample size, the roles of ICI plus anti-angiogenetic for were note classified, which was a promising strategy in EGFR-WT NSCLC.

For 20ins patients, previous studied reported that mPFS of first line chemotherapy, TKI, or ICI monotherapy in EGFR 20ins patients ranged 4.5–6.4, 0.7–3.7, or 3.1–4.3 months, respectively [4–8]. In POSITION20 trial, mPFS of Osimertinib in 20ins patients was 6.8 months and mOS was 15.2 months [30]. In PAPILLON study, the mPFS of first-line amivantamab plus chemotherapy was 11.4 months [31]. Our data revealed that ICI plus chemotherapy (mPFS of 8.30 months), but not ICI monotherapy, was superior to chemotherapy. ICI plus chemotherapy (mPFS of 9.13 months, mOS over 49.23 months) was also superior to ICI monotherapy in the Hong et al.’s cohort. Hence, for EGFR 20ins patients, ICI plus chemotherapy should be considered with more priority than chemotherapy or TKI.

For 19del/L858R patients, the IMpower 150 [20], ORIENT-31 [21] and ATTLAS [22] studies confirmed that ICI plus anti-angiogenetic and chemotherapy prolonged survival. Considering the drug accessibility and adverse effects, other optional regiments were needed. Here, we found that ICI plus chemotherapy prolonged PFS compared with chemotherapy alone. ICI plus anti-angiogenetic is a promising regimen for patients with EGFR WT, with a reported mPFS of 15.0 months [32] and 6.0 months [33]. However, in EGFR-mut patients, anlotinib plus ICI prolonged mPFS from 3.60 months of chemotherapy to only 4.33 months [34]. In the present study, the effectiveness of ICI plus anti-angiogenetic and chemotherapy was similar. Therefore, except for ICI plus chemotherapy and anti-angiogenetic, ICI plus chemotherapy, but not ICI plus anti-angiogenetic, is an effective option for 19del/L858R patients.

Our findings regarding the differences in TMB, but not PD-L1 expressions, across EGFR mutation subtypes may explain the heterogeneous responses to ICI since TMB is an important biomarker for ICI [13, 14]. However, TMB was not the only regulator, neoantigen quality and tumor microenvironment variability were also important. To explore the potential underlying mechanisms, we also found that uncommon EGFR mutations were associated with increased M1 macrophage infiltration and decreased Treg and M2 macrophage infiltration. A recent investigation found that macrophage infiltration differed between EGFR-mutant and WT lung adenocarcinoma, and some specific macrophage populations might regulate the response to ICI [35]. Another study found increased numbers of Tregs in EGFR-mutant brain metastases compared with EGFR WT [36]. Our previous study showed that EGFR T790M-*cis*-L792F induced M2 macrophage polarization [37]. Nevertheless, the molecular mechanisms by which specific EGFR mutation subtypes modulate macrophage polarization and Treg differentiation remain unclear. Considering the vital role of macrophage polarization and Tregs infiltrations, predicting response to ICI with them may better predict the response to ICI [38–41], compared to rely on PD-L1 expression only.

There were several limitations of this study. Firstly, the retrospective design and the relatively small sample size of patients when comparing different ICI strategy in specific EGFR mutation subtypes, especially a handful of patients receiving ICI + chemo + AA, lead to the limited statistical power. Secondly, it is necessary to further investigate the diverse response of each specific mutation within the uncommon group, such as G917X, S768I or L861Q. Due to the low mutation frequencies of them, large scale multicenter studies were expected. Thirdly, when analyzing tumor immune microenvironments (TIME), all RNA-sequencing data and samples used for IHC was treatment naïve. Immunologically colder TIME after EGFR TKI resistance [37, 42, 43] may also contribute to heterogenous responses to ICI. Lastly, detailed molecular mechanisms behind heterogenous responses of EGFR subtypes have not been clearly demonstrated, future studies with larger outcome datasets from other centers and functional studies of the clinical observations tested in murine modes of specific EGFR mutations were expected to increase the robustness of our preliminary findings.

To conclude, we found that LUAD with EGFR uncommon mutations represents a completely different subtype with better outcomes of immunotherapy, warmer TIME, and relatively higher TMB. For patients with EGFR uncommon mutations, ICI alone is as effective as plus chemotherapy. For patients with EGFR 20ins mutations, additional chemotherapy based on ICI was necessary. While for 19del/L858R patients, ICI plus chemotherapy was an alternative. Our results have important implications in the stratification and combination of ICI in patients with different subtypes of EGFR mutations.

## Materials and methods

### Immunotherapy cohorts

In total, from January 1, 2016 to December 1, 2024, 114 EGFR-mutant LUAD patients receiving anti-PD-(L)1 therapies at Fudan University Shanghai Cancer Center (FUSCC) were included in the FUSCC cohort. The inclusion criteria were as follows: 1. age ≥ 18 years, 2. advanced or recurrent LUAD confirmed by pathology, 3. harboring EGFR mutations confirmed by super amplification refractory mutation system (super-ARMS) or next-generation sequencing (NGS), 4. receiving anti-PD-(L)1 antibody therapy at least once, and 5. Radiologically evaluable according to Response Evaluation Criteria in Solid Tumors, version 1.1 (RECIST v1.1). Patients received concurrent radiotherapy with or without chemotherapy when receiving chemotherapy were ruled out. The characteristics of these 114 patients were listed in Table S1.

During the same period, a total of 137 patients from the Henan Cancer Hospital and Shanxi Province Cancer Hospital were included in the validation cohort. The inclusion criteria were the same as described above. The features of these 137 patients were listed in Table S2.

A total of 405 EGFR-mutant LUAD patients from nine open-access cohorts [13, 18, 44–50], who received anti-PD-(L)1 therapy with detailed survival were analyzed to further validate previous findings. These nine open-access cohorts were named according to the first authors and merged into a consolidated public cohort. Detailed information is provided in Table S3.

In FUSCC and the validation cohorts, responses were assessed every 4–6 weeks after the initial treatment until objective disease progression, with the CT and MRI scans independently evaluated by at least two qualified investigators. Partial response, disease progression, and stable disease were defined according to the RECIST v1.1. Progression-free survival were defined as the time from the initial treatment to the date of disease progression or death. Patients who had not progressed as of June 1, 2023, were recorded as censored. In the nine public cohorts, responses were assessed according to the descriptions in each published study.

Prior to anti-PD-(L)1 therapy, 57 patients in the discovery cohort and 81 in the validation cohort receiving chemotherapy with detailed survival records were considered self-controls when comparing the efficiency of various immunotherapy combination strategies.

This study was registered on ClinicalTrials.gov (NCT06164574). Institutional Review Board (IRB) approval and exemptions were obtained from each institution. Informed consent was not required per the IRB, as the data collected were anonymized. The study was approved by the Ethics Committee of Fudan University Shanghai Cancer Center (IRB-2008223-9), the Ethics Committee of Shanxi Province Cancer Hospital (KY2024052), and Institutional Ethics Committee of Henan Cancer Hospital affiliated to Zhengzhou University (Approval number 2017407). Written informed consent was obtained from all patients.

### Non-immunotherapy cohorts

To assess survival in patients without receiving immunotherapy, 3436 LUAD patients from FUSCC receiving radical resections followed by neither adjuvant EGFR tyrosine kinase inhibitors (TKIs) nor immunotherapy were analyzed. This cohort has not been reported before. Recurrence-free survival (RFS), and overall survival (OS) were assessed.

To investigate the TMB across TGFR mutation subtypes, two public NGS sequencing cohorts, namely the SYSU (*n* = 899) and the MSKCC (*n* = 1168) cohort [51, 52], were also analyzed.

Whole-exon sequencing data and bulk RNA-seq data from the TCGA LUAD cohort, East Asian cohort [53], and our cohort [54] were used to estimate the PD-L1 expression and infiltration of immune cells with CIBERSORTx [55].

### Mutational detecting and analysis

For the patients from FUSCC, EGFR mutations status was confirmed by Super ARMS and targeted NGS Analysis. Briefly, DNA from formalin fixed paraffin embedded tumor or ctDNA was extracted. EGFR hot spots (exons 18–22) was amplified and detected by a AmoyDX Super ARMS kit. Or, the OncoScreen Plus panel targeting whole exomes of 520 cancer-related genes, including EGFR, TP53, KRAS, etc., was applied for NGS, as previous reported [56–58]. The sequencing depth was 100 × for tumor tissue and 10,000 × for ctDNA.

For the patients from Henan Cancer Hospital and Shanxi Province Cancer Hospital, the EGFR mutations status was detected by their specific methods, including ddPCR, Super-ARMS and NGS.

For the SYSU cohort and MSKCC cohort [51, 52], TCGA-LUAD cohort, and the nine open-access immunotherapy cohorts [13, 18, 44–50], details of detecting EGFR mutations may be found in the methods sections of these articles.

### Immunohistochemistry (IHC)

Experiments using tumor samples were approved by the Ethics Committee of Fudan University Shanghai Cancer Center (IRB-2008223-9). All surgically resected specimen of untreated pulmonary adenocarcinoma, whose EGFR status were confirmed as described above, were collected from FUSCC for further experiments. As we previously reported, for PD-L1expression assessment, the tumor proportion score (TPS) based on immunohistochemistry was conducted using validated monoclonal anti-PD-L1 antibody 22C3 (Dako North America Inc., Carpinteria, CA) using the Dako Autostainer Link 48 platform following its manufacturer’s instructions. TPS of PD-L1 expression were defined as the percentage of tumor cells with positive PD-L1 staining over all tumor cells. Specimens containing less than 100 measurable tumor cells were excluded. PD-L1 expression was further categorized into TPS < 1%, 1–49%, ≥ 50%. For immune cell infiltration assessments, paraffin-embedded sections were blocked with goat serum and stained with a primary antibody using an IHC staining kit (Zsbio, Beijing, China). Primary anti-CD8A (85336S), anti-FOXP3 (98377S), anti-CD86 (91882S), and anti-CD206 (91992S) were purchased from Cell Signaling Technology. Cell nuclei were stained with hematoxylin as previously reported [59]. Each sample was stained on a single slide and scanned at 200 × resolution. A region of interest (ROI) was defined as a field of view under 200 × resolution for each slide and used for quantitative analysis. Any slide containing less than 1000 cancer cells, or any ROI containing less than 20 cells would not be applied. Within each ROI, the number of specific cells was calculated based on positive biomarker expression. All ROIs were reviewed by two experienced pulmonary pathologists. Any disagreements were resolved by re-review and discussion until agreements were reached.

### Statistical analysis and graphics

Continuous variables (e.g., TMB) were compared using the *t*-test or Mann–Whitney *U* test. The significance of categorical variables (e.g., PD-L1 expression) was evaluated using the chi-square test or Fisher’s exact test. Survival was compared using Kaplan–Meier curves, and *p*-values were calculated using the log-rank test. Hazard ratios (HR) were determined using univariate or multivariate Cox regression analyses. *p* < 0.05 was considered statistically significant. When multiple comparisons were conducted, false discovery rate (FDR) based on the Benjamin-Hochberg approach was employed to adjust the statistical tests. FDR-q value < 0.05 was considered statistically significant. With GraphPad (version 8.0.3, San Diego, CA, USA) or R software (version 4.0.3, R Foundation for Statistical Computing, Vienna, Austria), all data were analyzed and all graphs were drawn.

## Supplementary Information

Supplementary Material 1: Fig. S1. Recurrence free survival and overall survival of surgery of EGFR mutation subtypes. (a-b). RFS (a) and OS (b) across EGFR mutation subtypes in 3436 patients harboring EGFR mutations received radical resection.

## Data Availability

The RNA and whole-exome sequencing data of TCGA LUAD were downloaded from the UCSC Xena platform (https://xenabrowser.net/datapages/) on December 7th, 2022. The data of east Asian cohort could be downloaded from cBioPortal (cbioportal.org). The data of our own cohort coud be downloaded according to the instruction in the article. NGS and clinical data including survival of these nine public cohorts could be obtained according to the instructions in the corresponding articles. Due to the regulations of the institutions, individual-level data of this study cannot be uploaded to the public repository, but available from the corresponding author upon reasonable requests. The scripts and bioinformatics code are available now on github (https://github.com/duanmuseventeen/sun-lab).
