# Supplementary material for: Distinct survival, optimal combination strategy of immunotherapy, and immunophenotype in uncommon and 20ins EGFR-mut lung adenocarcinoma: a multi-center study
Source: Mol Biomed. 2025 Oct 30;6:88. doi: 10.1186/s43556-025-00331-1 (PMC12572489; doi:10.1186/s43556-025-00331-1)
Supplement: Supplementary file 1 — Supplementary Material 1: Fig. S1. Recurrence free survival and overall survival of surgery of EGFR mutation subtypes. (a-b). RFS (a) and OS (b) across EGFR mutation subtypes in 3436 patients harboring EGFR mutations received radical resection. [file 43556_2025_331_MOESM1_ESM.docx]

**Distinct survival, optimal combination strategy of immunotherapy, and immunophenotype in uncommon and 20ins EGFR-mut lung adenocarcinoma: a multi-center study**

Yiting Sun^1,2,3†^, Lei Xu^4,5†^, Chaoqiang Deng^1,2,3†^, Xinyang Du^6†^, Yongkui Yu^7^, Yuan Hao^8^, Huijuan Wang^6*^, Xin Wang^9,8*^, Yang Zhang^1,2,3*^, and Haiquan Chen^1,2,3*^

1. Departments of Thoracic Surgery and State Key Laboratory of Genetic Engineering, Fudan University Shanghai Cancer Center, Shanghai, China

2. Institute of Thoracic Oncology, Fudan University, Shanghai, China

3. Department of Oncology, Shanghai Medical College, Fudan University, Shanghai, China

4. Departments of Thoracic Surgery, Shanghai Chest Hospital, School of Medicine，Shanghai Jiao Tong University, Shanghai, China.

5. Department of Thoracic Surgery, National Cancer Center/National Clinical Research Center for Cancer/Cancer Hospital, Chinese Academy of Medical Sciences and Peking Union Medical College, Beijing, China

6. Department of Medical Oncology, The Affiliated Cancer Hospital of Zhengzhou University & Henan Cancer Hospital, Zhengzhou, Henan Province, China

7. Department of Thoracic Surgery, The Affiliated Cancer Hospital of Zhengzhou University & Henan Cancer Hospital, Zhengzhou, Henan Province, China

8. Department of Clinical Trials Center, Shanxi Province Cancer Hospital/Shanxi Hospital Affiliated to Cancer Hospital, Chinese Academy of Medical Sciences/Cancer Hospital Affiliated to Shanxi Medical University, Taiyuan, Shanxi Province, China

9. Department of Clinical Trials Center, National Cancer Center/National Clinical Research Center for Cancer/Cancer Hospital, Chinese Academy of Medical Sciences and Peking Union Medical College, Beijing, China

† Y. Sun, L. Xu, C. Deng, and X. Du contributed equally as co-first authors of this article.

* Corresponding Authors: Haiquan Chen (hqchen1@yahoo.com), Yang Zhang ([fduzhangyang1987@hotmail.com](mailto:fduzhangyang1987@hotmail.com)), Xin Wang ([xiaowuxian2006@126.com](mailto:xiaowuxian2006@126.com)), and Huijuan Wang ([18638561588@163.com](mailto:18638561588@163.com)).

**Supplementary figures and figure legends**

Fig. S1. Recurrence free survivals and overall survivals of surgery of EGFR mutation subtypes.


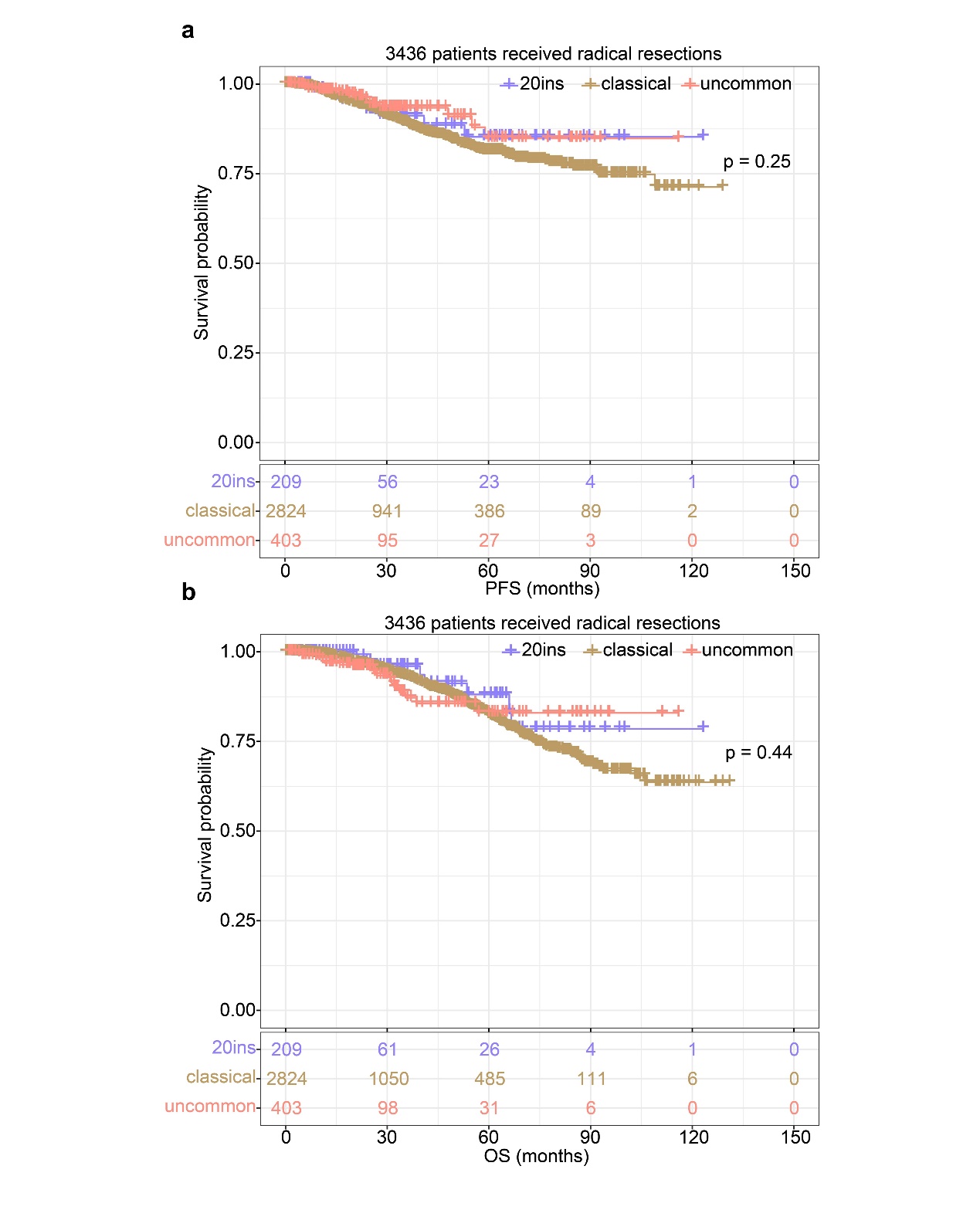


(a-b). RFS (a) and OS (b) across EGFR mutation subtypes in 3436 patients harboring EGFR mutations received radical resection. RFS, recurrence-free survivals; OS, overall survivals.

**Supplementary Tables.**

Table S1. Characteristics of patients with EGFR mutations treated with immune checkpoint inhibitors in the discovery cohort.

|  | EGFR mutation subtypes | | |  |
| --- | --- | --- | --- | --- |
|  | uncommon | classical | 20ins | p value |
| all | 45 | 47 | 22 |  |
| Smoking History |  |  |  |  |
| Ever | 30 | 16 | 12 |  |
| Never | 5 | 18 | 5 |  |
| N.A. | 10 | 13 | 5 | 0.016 |
| Lines of therapy |  |  |  |  |
| 1st | 5 | 1 | 7 |  |
| 2nd | 27 | 19 | 11 |  |
| 3+lines | 13 | 27 | 4 | 0.0003 |
| Drug Target |  |  |  |  |
| PD-1 | 45 | 46 | 20 |  |
| PD-L1 | 0 | 1 | 2 | 0.089 |
| Treatment Strategy |  |  |  |  |
| ICIs monotherapy | 16 | 12 | 7 |  |
| ICIs plus other therapy | 29 | 35 | 15 | 0.57 |
| Progression-Free Survivals | |  |  |  |
| Median | 10.27 | 4.4 | 3.80 |  |
| Range | 0.70-28.33 | 0.47-45.77 | 1.03-27.3 |  |
| Best response |  |  |  |  |
| PR | 33 | 17 | 6 |  |
| SD | 5 | 14 | 6 |  |
| PD | 7 | 16 | 10 | 0.0010 |
| PD-L1 expression |  |  |  |  |
| <1% | 12 | 11 | 6 |  |
| >1% | 23 | 17 | 6 |  |
| N.A. | 10 | 19 | 10 | 0.22 |

N.A., not available; ICI, immune checkpoint inhibitors; PR, partial response; SD, stable disease; PD, progression disease; TKI, tyrosine kinase inhibitors.

Table S2. Characteristics of patients with EGFR mutations treated with immune checkpoint inhibitors in the validation cohort.

|  | EGFR mutation subtypes | | |  |  |
| --- | --- | --- | --- | --- | --- |
|  | uncommon | classical | 20ins | all | p value |
| All | 46 | 53 | 38 | 137 |  |
| Smoking History |  |  |  |  | 0.026 |
| Ever | 24 | 14 | 21 | 59 |  |
| Never | 19 | 34 | 16 | 59 |  |
| N.A. | 3 | 5 | 1 | 9 |  |
| Lines of therapy |  |  |  |  | <0.0001 |
| 1st | 10 | 0 | 14 | 25 |  |
| 2nd | 17 | 12 | 16 | 44 |  |
| 3+lines | 19 | 41 | 8 | 58 |  |
| Drug Target |  |  |  |  | 0.14 |
| PD-1 | 43 | 53 | 37 | 133 |  |
| PD-L1 | 3 | 0 | 1 | 4 |  |
| Treatment Strategy |  |  |  |  | 0.55 |
| ICI monotherapy | 12 | 17 | 14 | 43 |  |
| ICI plus other therapy | 34 | 36 | 24 | 94 |  |
| Progression-Free Survivals | |  |  |  |  |
| Median | 17.2 | 6.47 | 9.43 |  |  |
| Range | 0.93-44.53 | 0.47-30.67 | 1.40-46.80 | |  |
| Best response |  |  |  |  | 0.0051 |
| PR | 34 | 23 | 26 | 83 |  |
| SD | 9 | 13 | 8 | 30 |  |
| PD | 3 | 17 | 4 | 24 |  |
| EGFR TKI before ICI |  |  |  |  | p<0.0001 |
| Yes | 14 | 53 | 7 | 74 |  |
| No | 32 | 0 | 31 | 63 |  |
| PD-L1 expression |  |  |  |  | 0.13 |
| <1% | 3 | 7 | 4 | 14 |  |
| >1% | 25 | 16 | 19 | 60 |  |
| N.A. | 18 | 30 | 15 | 63 |  |

N.A., not available; ICI, immune checkpoint inhibitors; PR, partial response; SD, stable disease; PD, progression disease; TKI, tyrosine kinase inhibitors.

| cohort | samples | sequencing methods | survivals | EGFR-mut patients | drugs |
| --- | --- | --- | --- | --- | --- |
| Hong et al. | ctDNA  or tissue | panel | PFS+OS | 96 | ICI mono  / combo |
| Hasting et al. | N.R. | N.R. | PFS+OS | 171 | ICI mono |
| Gandara et al. | ctDNA | panel | PFS+OS | 35 | ICI mono |
| Rizvi et al. | tissue | panel | PFS | 24 | ICI mono |
| Vanguri et al. | tissue | panel | PFS+OS | 16 | ICI mono |
| Hellmann et al. | tissue | WES | PFS | 8 | ICI mono |
| Samstein et al. | tissue | panel | OS | 39 | ICI mono |
| Anasnostou et al. | tissue | WES | OS | 5 | ICI mono |
| Miao et al. | tissue | WES | PFS+OS | 11 | ICI mono |

Table S3. Features of nine open access cohorts.

PFS, progression-free survivals; OS, overall survivals; ICI, immune checkpoint inhibitors; WES, whole-exon sequencing. N.R., not recorded.

Table S4. Overview of survivals across the three mutation groups for each treatment strategy.

|  | uncommon | | | classical | | 20ins | |
| --- | --- | --- | --- | --- | --- | --- | --- |
|  | our cohorts | Hong et al.’s | Gandara et al.'s | our cohorts | Gandara et al.'s | our cohorts | Hong et al.’s |
| chemo-PFS | 6.3 |  | 3.02 | 4.75 | 4.17 | 5.67 |  |
| ICI-mono-PFS | 15.3 | 6.73 | 3.88 | 4.10 | 1.68 | 5.67 | 3.40 |
| ICI+chemo-PFS | 21.37 | 8.30 |  | 9.17 |  | 8.06 | 9.13 |
| ICI+AA-PFS | 16.37 |  |  | 3.07 |  | 11.60 |  |
| ICI+AA+chemo-PFS | 26.63 |  |  | 11.12 |  | 16.67 |  |
| chemo-OS |  |  | 9.12 |  | 15.31 |  |  |
| ICI-mono-OS |  | 23.75 | 15.67 |  | 9.50 |  | 25.30 |
| ICI+chemo-OS |  | 28.97 |  |  |  |  | 49.23 |
| ICI+AA-OS |  |  |  |  |  |  |  |
| ICI+chemo+AA-OS |  |  |  |  |  |  |  |

AA, anti-angiogenic therapies.
